# Reduction of metal artifacts from knee tumor prostheses on CT images: value of the single energy metal artifact reduction (SEMAR) algorithm

**DOI:** 10.1186/s12885-021-09029-3

**Published:** 2021-12-02

**Authors:** Fang-ling Zhang, Ruo-cheng Li, Xiao-ling Zhang, Zhao-hui Zhang, Ling Ma, Lei Ding

**Affiliations:** Department of Radiology, The First Affiliated Hospital, Sun Yat-sen University, 58# Zhongshan Er Road, 510080 Guangzhou, Guangdong Province People’s Republic of China

**Keywords:** Metal artifact reduction, Tumor prosthesis, Knee, Computed tomography, Image quality

## Abstract

**Background:**

To evaluate the effect of the single energy metal artifact reduction (SEMAR) algorithm with a multidetector CT (MDCT) for knee tumor prostheses.

**Methods:**

First, a phantom of knee tumor prosthesis underwent a MDCT scan. The raw data was reconstructed by iterative reconstruction (IR) alone and IR plus SEMAR. The mean value of the CT number and the image noise were measured around the prosthesis at the stem level and articular level. Second, 95 consecutive patients with knee tumor prostheses underwent MDCT scans. The raw data were also reconstructed by the two methods. Periprosthetic structures were selected at the similar two levels. Four radiologists visually graded the image quality on a scale from 0 to 5. Additionally, the readers also assessed the presence of prosthetic complication and tumor recurrence on a same scale.

**Results:**

In the phantom, when the SEMAR was used, the CT numbers were closer to normal value and the noise of images using soft and sharper kernel were respectively reduced by up to 77.1% and 43.4% at the stem level, and by up to 82.2% and 64.5% at the articular level. The subjective scores increased 1 ~ 3 points and 1 ~ 4 points at the two levels, respectively. Prosthetic complications and tumor recurrence were diagnosed in 66 patients. And the SEMAR increased the diagnostic confidence of prosthetic complications and tumor recurrence (4 ~ 5 vs. 1 ~ 1.5).

**Conclusions:**

The SEMAR algorithm can significantly reduce the metal artifacts and increase diagnostic confidence of prosthetic complications and tumor recurrence in patients with knee tumor prostheses.

## Key points

• The SEMAR algorithm can significantly reduce artifacts caused by knee tumor prostheses.
• The SEMAR algorithm helps to assess prosthetic complications and tumor recurrence.

## Background

Primary malignant bone tumors are most frequently located in the distal femur and proximal tibia [1]. Tumor prosthesis is commonly used to reconstruct the knee joint in limb salvage surgery. Although the survival rate of patients is currently satisfactory [2–4], there are still some potentially serious complications, including prosthetic osteolysis, breakage, infection, periprosthetic fracture, and especially tumor recurrence, meaning that radiologists have to provide a more accurate evaluation of periprosthetic structures and lesions. Nevertheless, the knee tumor prosthesis consists of substantial high-density metal alloy, which can produce extensive artifacts on CT images because of scattering, photon starvation and x-ray beam hardening [5], which can superimpose upon other structures and result in missed diagnosis of prosthetic complication. Various methods have been introduced to reduce the metal artifacts, including higher peak voltage, higher tube charge, MAR algorithms, the dual-energy CT techniques [6]. However, higher peak voltage and tube charge may only reduce metal artifacts on a minor degree and lead to a higher radiation dose to the patient. Therefore, CT with metal artifact reduction (MAR) and dual-energy techniques are currently used to reduce the metal artifacts.

Up to now, the MAR algorithms generally can be categorized into IR methods and interpolation-based methods [7]. The IR methods require multiple forward and backward projections, which need computational cost is too high in the past few years. With the available computational power and techniques improving, IR methods can be clinically applied nowadays. However, when IR methods were used alone, although the image quality was improved [8], the metal artifacts remain greatly hampering the visualization of periprosthetic structures [9]. The interpolation-based methods are to replace metal-corrupted projection data with surrogate data from interpolation using surrounding uncorrupted sinogram information [7, 10]. However, when it comes to large metal implants, the reliability of many pure interpolation-based methods decreases considerably [7]. Therefore, overcoming the metal artifacts of tumor prostheses in post-surgery follow-up is still a challenge [5].

Recently, several commercial MAR software which work on projection–interpolation methods have been proposed. The single-energy metal artifact reduction (SEMAR, Canon Medical Systems) is one of the projection-based metal reduction algorithms, which was clinically introduced on a second-generation 320-row CT scanner (Aquilion ONE, Canon Medical Systems) [11]. Previous studies using the SEMAR algorithm have shown an improvement of image quality in patients with metallic implants, such as aneurysm embolization coils, hip prostheses, dental hardware, and so on [9–18]. The usefulness of SEMAR in patients with knee tumor prostheses has not yet been established, and there were no literature referring the metal artifact reduction of knee tumor prosthesis, which may be the thickest and densest metal implant in human body. In this investigation, we compared the image quality with IR algorithm alone and in association with SEMAR algorithm in a phantom and patients with modular knee tumor prostheses, and tried to ascertain the effect of SEMAR in the quality of images and diagnostic workup of prosthetic complications and tumor recurrence.

## Materials and methods

Our institutional review board approved this clinical study, and informed consent was obtained from all patients. All methods in this study were in accordance with relevant guidelines and regulations of our hospital.

### Phantom

The first part of this study was a phantom experiment. A modular rotating-hinge knee tumor prosthesis (Beijing Lidakang Technology Co., Ltd) was placed in a water-filled plastic case, in which the prosthesis was fixed in another plastic case to keep it in the center of the phantom. The two cases had a cross-section of 35 × 30 cm^2^ and 25 × 20 cm^2^, respectively. The phantom was filled with water to a depth of 20 cm with water. The knee tumor prosthesis consists of distal femoral and proximal tibial component, including a modular rotating hinge knee, cemented stem, and extension pieces. The articular part was made of cobalt-chrome-molybdenum alloy and the stem was made of titanium alloy for strength and light weight.

After the phantom was scanned on CT, the phantom was kept unmoved, and the knee prosthesis was removed from the phantom, and added water into the cases to keep the same depth of 20 cm. Then the phantom without prosthesis was scanned at the same parameters to determine the “true” Hounsfied units of water for this setting.

### Patients

The second part of the study was performed by using the radiologic database from November 2015 to October 2017 in our hospital. We reviewed the plain and enhanced CT images of the knee joint performed on 95 consecutive patients (59 males, 36 females; mean age, 24.2 years; age range, 9–64 years) with modular rotating-hinge knee tumor prostheses retrospectively (Beijing Lidakang Technology Co., Ltd). The patients’ tumors consisted of 75 osteosarcomas, 14 giant cell tumors, 2 Ewing’s sarcomas, 2 myogenic sarcomas, 1 chondrosarcoma and 1 undifferentiated pleomorphic sarcoma, 65 of which were located in the distal femur and 30 in the proximal tibia. The right side was involved 49 times, and the left side was involved 46 times. Their personal information was anonymized for evaluation.

### Data acquisition

All CT examinations were performed with a 320-row multidetector CT scanner (MDCT). The axial scan parameters were: scan mode, volumetric; tube voltage, 135 kV; tube current, automatic exposure control (SURE exposure 3D, Canon Medical Systems); detector collimation, 320 × 0.5 mm; gantry rotation time, 1.0 s; and matrix 512 × 512. Contrast agent with an iodine concentration of 300 mg/ml (Ultravist 300, Bayer AG) were used in all the patients after plain scan. The total volume of contrast material (ml) was determined by multiplying the body weight (kg) by two, with an upper limit of 100 ml. It was injected at a rate of 2.5 ml/s via a 22-gauge intravenous catheter placed in an antecubital vein. The enhanced CT scan began 70 s after the initiation of contrast injection.

### Image reconstruction

The adaptive iterative reconstruction (IR) algorithm (AIDR 3D, Canon Medical Systems) and IR plus SEMAR algorithm (version 7.0) were applied to the raw data. In our hospital’s clinic for CT examinations, a standard soft kernel (FC08) is usually used for depiction of soft tissues and a sharper kernel (FC30) for examination of bone. Therefore, both the soft tissue kernel and sharper kernel were used for the two reconstructions. The SEMAR algorithm automatically removed metal artifacts according to various steps of data segmentation, forward projection, interpolation, and back projection, which has been reported previously [9, 12].

### Image assessment

#### Objective evaluation

**Fig. 1:**
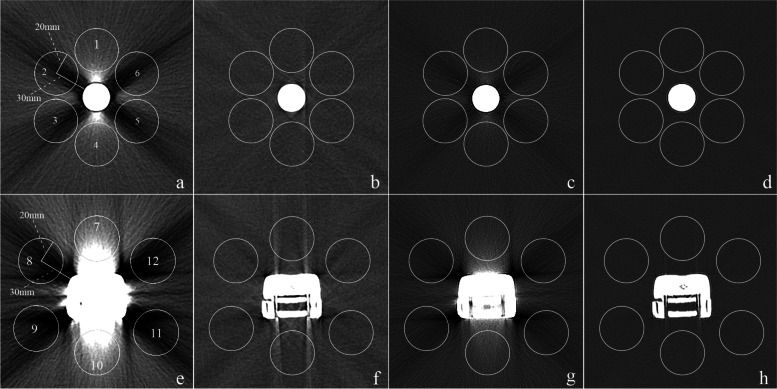
ROIs in the CT images of phantom. ROIs were placed 3 cm form the prosthesis and kept consistent on non-SEMAR images and SEMAR images. At the stem level (**a** ~ **d**) and articular level (**e** ~ **h**), axial non-SEMAR image with soft kernel (**a**, **e**) and sharper kernel (**c**, **g**) reveals prominent, sharp, streak artifacts, while axial SEMAR image with soft kernel (**b**, **f**) and sharper kernel (**d**, **h**) demonstrating markedly reduced artifacts

As the articular part of knee tumor prosthesis was dense and made of cobalt-chrome-molybdenum alloy with a high atomic number (atomic number 42 for molybdenum, 27 for cobalt and 24 for chromium), producing heavy artifacts, while the stem was relatively small and made of titanium with a relatively low atomic number of 22, producing minor artifacts [6], the images of phantom were evaluated at two levels: the stem level and the articular level. A board-certified radiologist with 8 years of clinical experience measured the CT numbers (in Hounsfield units [HU]) using a circular region of interest (ROI) with a diameter of 4 cm, and 6 ROIs were selected at each level and were kept 3 cm away from metal mass on non-SEMAR and SEMAR images (Fig. 1). On the images of phantom without prosthesis, the ROIs were also placed at the same position as before to get the CT value of water for this setting. The absolute measurement error of CT values of the ROIs with and without prosthesis was evaluated, which is closer to “zero” meaning the CT values closer to the true values of the water for this setting. Image noise was defined as the standard deviation (SD) of CT numbers in HU.

Subjective evaluation.

**Fig. 2:**
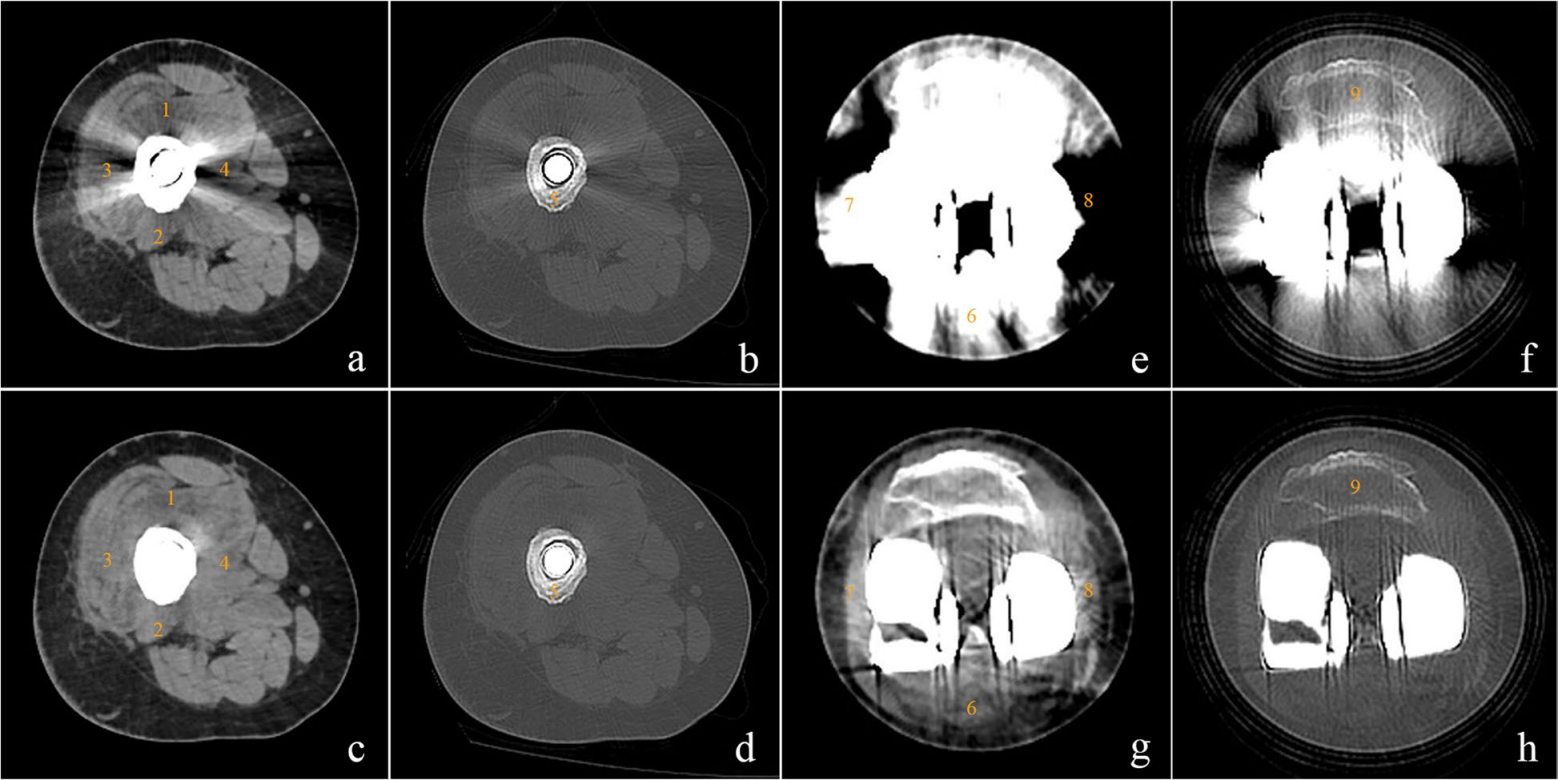
The regions of interest (ROIs) around a proximal femur tumor prosthesis at the osteotomy level (**a**, **b**, **c**, **d**) and articular level (**e**, **f**, **g**, **h**). 9 ROIs were selected around the prosthesis: (1) ~ (4) the muscles surrounding the prosthesis stem (**a**, **c**), (5) the periprosthetic bone at the osteotomy level (**b**, **d**), (6) ~ (8) the muscles or tendons surrounding the articular part of the prosthesis (**e**, **g**) and (9) the patella at the articular level (**f**, **h**). The window width and level were 400/40 HU (**a**, **c**, **e**, **g**) and 2200/400 HU (**b**, **d**, **f**, **h**), respectively. On non-SEMAR images (**a**, **b**, **e**, **f**), the prosthesis produces extensive metal artifacts, especially at the articular level. The SEMAR reconstruction (**c**, **d**, **g**, **h**) considerably reduces the dark and sharp streak artifacts

The images of patients were subjectively evaluated at two levels: the osteotomy level and the articular level. 9 ROIs were selected on unenhanced CT images (Fig. 2): (1) ~ (4) the muscles surrounding the prosthesis stem, (5) the periprosthetic bone at the osteotomy level, (6) ~ (8) the muscles or tendons surrounding the articular part of the prosthesis and (9) the patella at the articular level. Four board-certified musculoskeletal radiologists with 8 to 15 years of clinical experience assessed the images independently and blindly. Images with different reconstruction methods were showed on a high-resolution 20-inch monitor (M21, Nanjing Jusha Commercial &Trading Co., Ltd) individually and randomly. For soft tissue evaluation, all the images were reconstructed by a standard soft tissue kernel (FC08) and evaluated in the axial plane using a 40/400 HU window width/level setting. Meanwhile, for bone evaluation, the images were reconstructed by a standard bone kernel (FC30) and analyzed using a window width/level of 400/2200 HU. The visualization of periprosthetic anatomic structures on the images was graded as follows: 0 = periprosthetic anatomic structure completely obscured; 1 = marked artifacts with questionable recognition of periprosthetic anatomic structure; 2 = faint anatomic recognition; 3 = recognition with low confidence; 4 = recognition with medium confidence; 5 = recognition with high confidence [9, 19, 20]. Additionally, the readers evaluated the prosthetic complications and tumor recurrence on enhanced images. If present, lesions were rated for diagnostic confidence by the same scoring system.

### Statistical analysis

All statistical analyses were performed using the statistical software package SPSS25.0 (SPSS Inc.). The objective image quality data of the phantom was expressed as the mean ± SD, and compared using the student’s paired t-test. The scores of subjective image quality were expressed as median ± interquartile range, which were compared by the Wilcoxon matched-pairs signed rank test. Intra-class correlation coefficients (ICCs) were calculated to assess inter-observer variability. Different guidelines exist for the interpretation of ICC, but one reasonable scale is that an ICC value of 0.40 or less indicates poor agreement; 0.41–0.59 indicates fair agreement; 0.60–0.74 indicates good agreement; and 0.75–1.00 indicates excellent agreement [21]. A *P* value less than 0.05 was considered statistically significant.

## Results

### Image assessment

Objective evaluation.

**Fig. 3:**
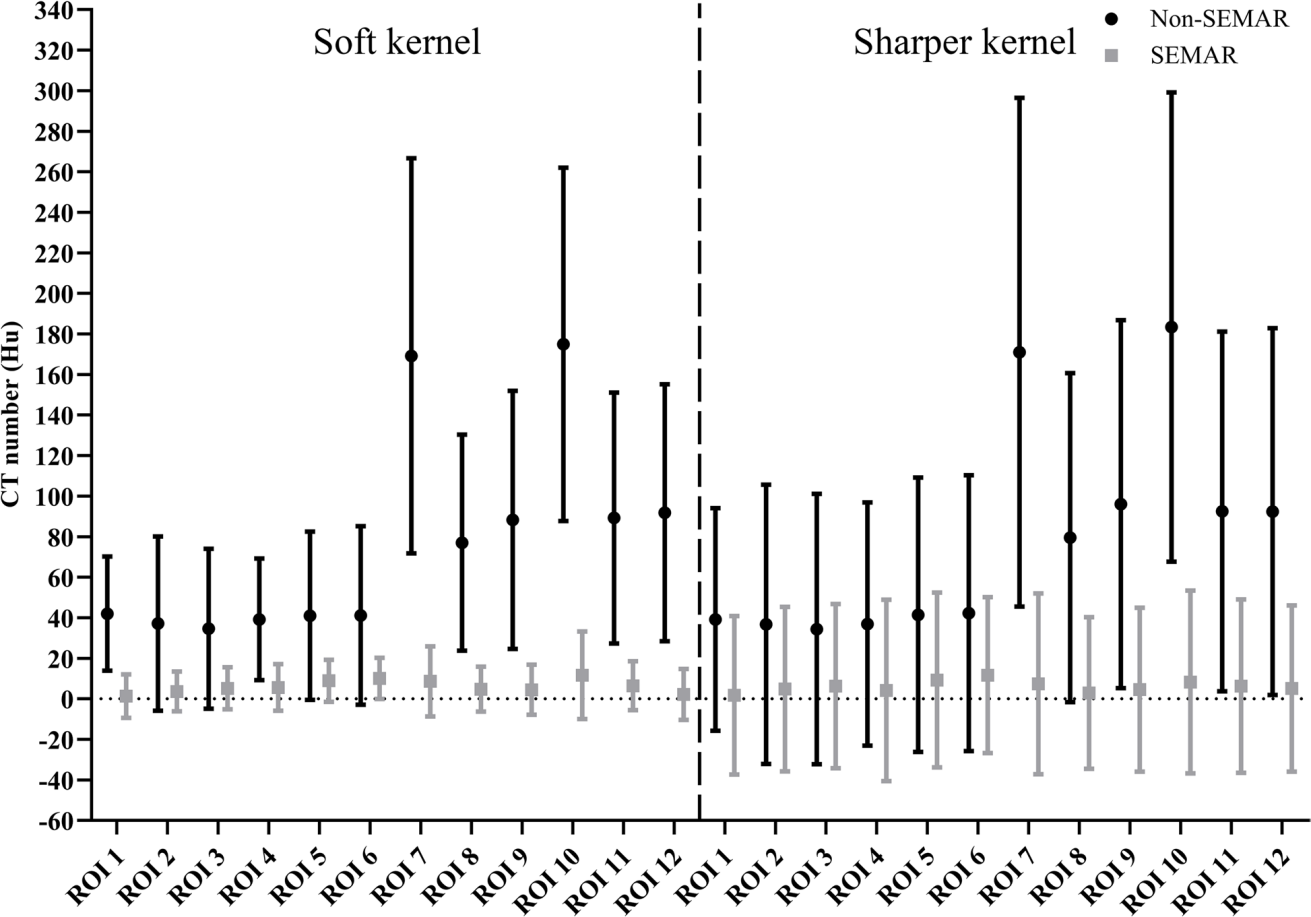
The absolute measurement error and the noise (standard deviation) of the CT numbers in the ROIs (marked in Fig. 1) in CT images at the stem level and articular level of the phantom. The values are shown for the different reconstruction settings: soft/sharper kernel and non-SEMAR/SEMAR reconstruction

The results of the phantom images were showed in Fig. 3, which show that when the SEMAR was used, the absolute measurement error of CT values with and without prosthesis were closer to zero and the image noise was obviously reduced in the ROIs at both the articular and stem levels. The results of soft kernel were as follows [SEMAR vs. non-SEMAR (CT number ± noise)]: articular level, 2.11 ~ 11.64 ± 11.08 ~ 21.59 Hu vs. 77.01 ~ 174.90 ± 53.27 ~ 97.41Hu (*P* < 0.001); stem level, 1.34 ~ 10.11 ± 9.82 ~ 11.46 vs. 34.58 ~ 42.03 ± 28.15 ~ 44.09 (*P* < 0.001). While for sharper kernel, the results were (SEMAR vs. non-SEMAR): Articular level, 2.87 ~ 8.31 ± 37.40 ~ 45.15 Hu vs. 79.49 ~ 183.41 ± 81.18 ~ 125.49Hu (*P* < 0.001); Stem level, 1.75 ~ 11.67 ± 38.50 ~ 44.78 vs. 34.40 ~ 42.30 ± 54.89 ~ 68.90 (*P* < 0.001). When the SEMAR was used, the noises of ROIs on the CT images using soft kernel and the sharper kernel were respectively reduced by up to 77.1% and 43.4% at the stem level, 82.2% and 64.5% at the articular level (*P* < 0.001).

Subjective evaluation.

**Fig. 4:**
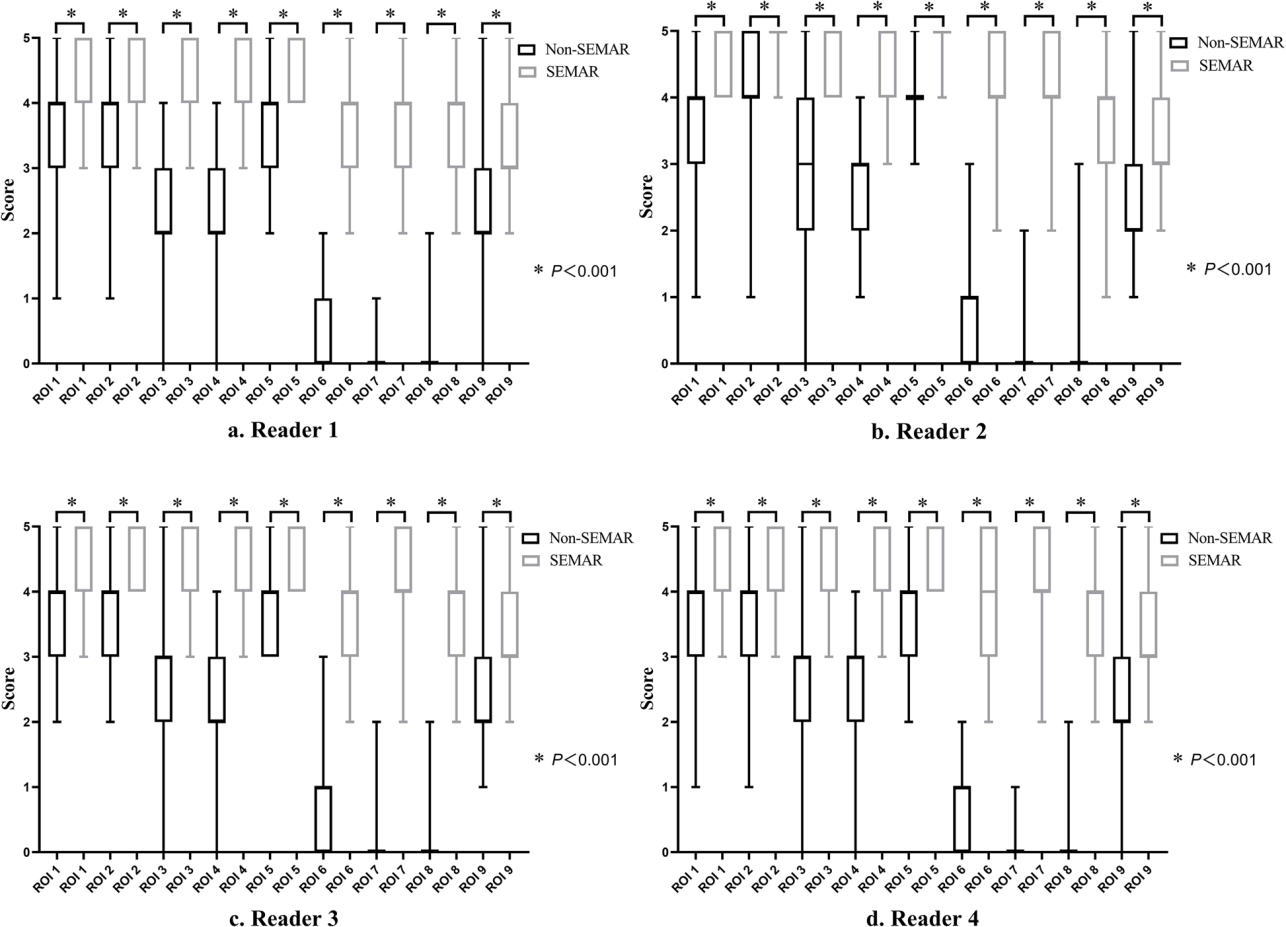
Boxplot showing the scores of image quality by four readers (a-d). Better visual scores of all ROIs were obtained with SEMAR (3 ~ 5 vs. 0 ~ 4, *P* < 0.001)

The substantial metal artifacts from the tumor prostheses made the recognition of the periprosthetic structures very challenging without SEMAR, especially at the articular level (Fig. 2). Nevertheless, visualization was significantly improved for all readers when the SEMAR algorithm was used (3 ~ 5 vs. 0 ~ 4, *P* < 0.001) (Fig. 4). The subjective scores of the images at the stem level and articular level increased 1 ~ 3 scores and 1 ~ 4 scores, respectively. Interobserver agreement was considered to be excellent with both reconstruction types (ICC = 0.98).

### The abnormalities in patients

**Fig. 5:**
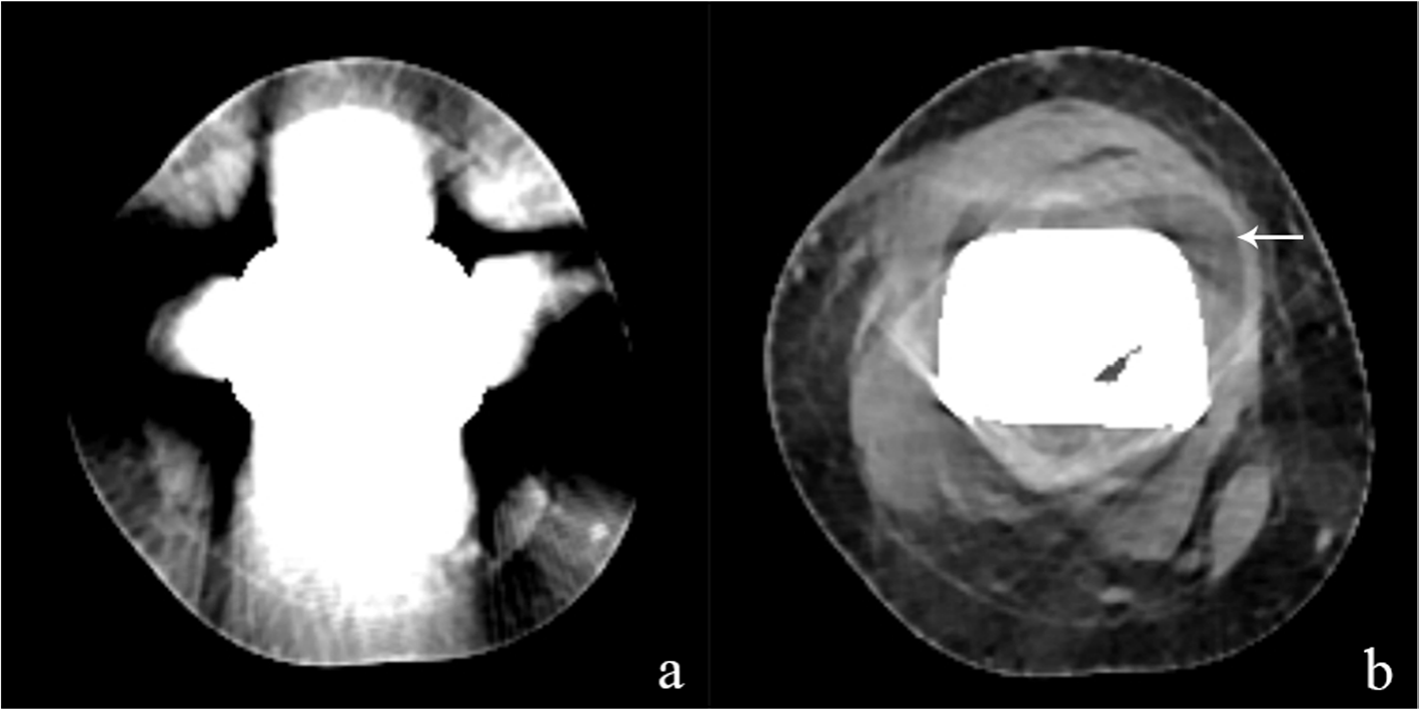
The unenhanced CT images of a 22-year-old woman with a distal femur tumor prosthesis show periprosthetic structures were almost obscured by metal artifacts on the IR image (**a**), which are visibly eliminated on SEMAR image, and the periprosthetic effusion (arrow) appears

**Fig. 6:**
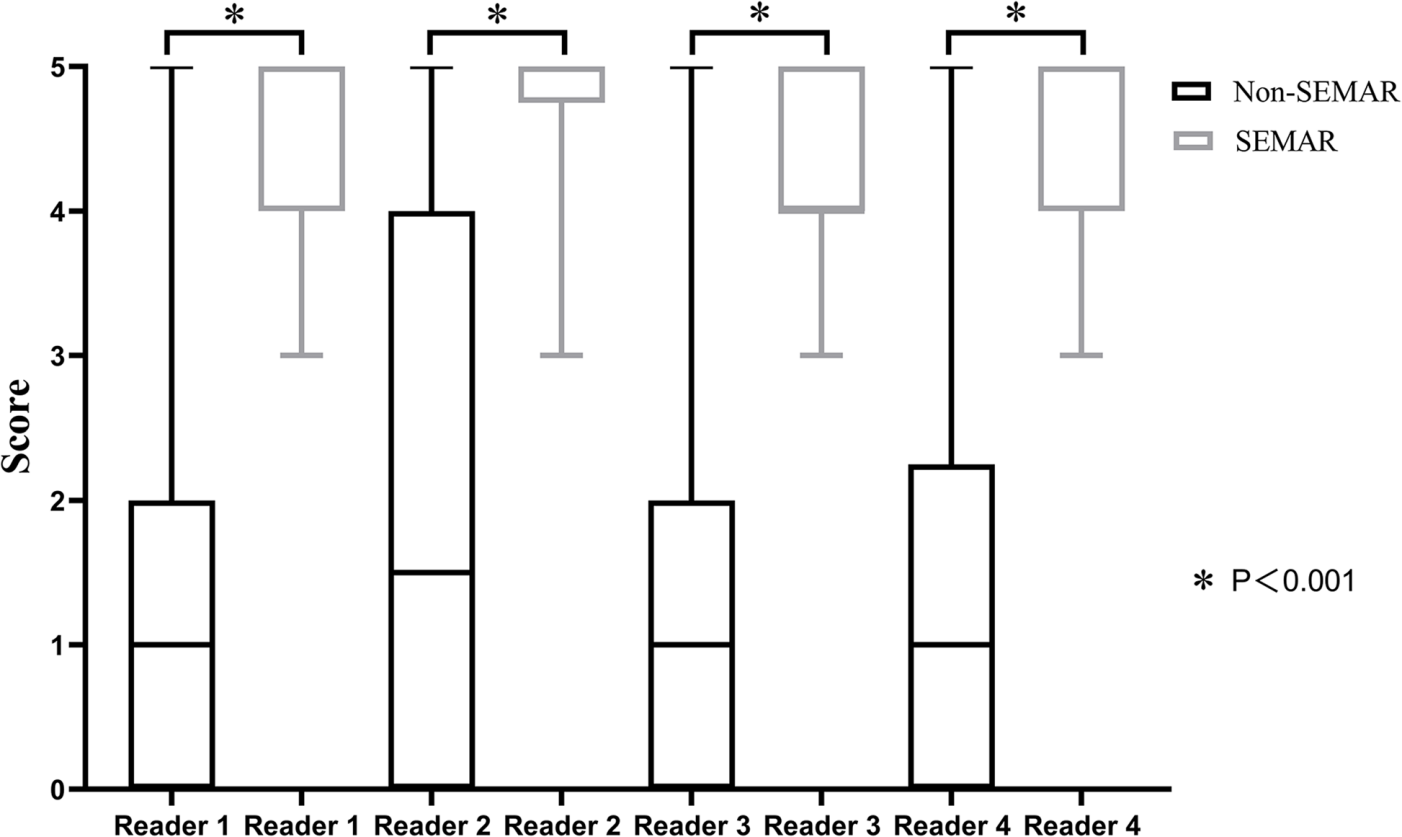
Boxplot showing the scores of prosthetic complications by four readers. The SEMAR significantly increased diagnostic confidence of prosthetic complications (4 ~ 5 vs. 1 ~ 1.5, *P* < 0.001)

Abnormalities were confirmed in 66 patients by other imaging/pathology examination or clinical follow-up, including periprosthetic effusion in 44 patients (Fig. 5), prosthetic osteolysis in 8 patients, periprosthetic fracture in 5 patients, and tumor recurrence in 9 patients. The SEMAR algorithm significantly increased the diagnostic confidence of lesions (4 ~ 5 vs. 1 ~ 1.5, *P* < 0.001) (Fig. 6). Interobserver agreement was considered to be excellent with both reconstruction types (ICC = 0.97).

## Discussion

The metal implants in human body can produce artifacts on CT images because of scattering, photon starvation and x-ray beam hardening [5]. At the energy levels used for diagnostic imaging, the photoelectric effect is proportional to the cube of the atomic number of the metal implants [6]. The articular part of the knee tumor prosthesis was made of metal with a high atomic number (cobalt-chrome-molybdenum alloy), creating photon-starved effect more severely [6]. Therefore, there were maximal streak artifacts visually at the articular level. When using SEMAR, the CT number accuracies of the phantom were improved on images with both soft kernel and sharper kernel, and the noises were obviously decreased by up to 82.2% and 64.5% (*P* < 0.001). On the CT images of patients, the structures near the prostheses were prominently obscured when using adaptive IR alone. The periprosthetic soft tissue evaluated was at best questionably recognized (median scores 0 ~ 1), and the patella was at best faint recognized (median scores 2). The use of the SEMAR algorithm upgraded the image quality obviously (*P* < 0.001). Periprosthetic soft tissue and the patella was recognized with low to medium confidence (median scores 3–4).

At the osteotomy level, the stem was made of metal with a lower atomic number (titanium), which may only cause minor beam hardening [6]. Therefore, the metal artifacts were not so obvious as at the articular level. Nevertheless, the absolute measurement error of CT values on images with both soft kernel and sharper kernel in the phantom were also closer to zero, and the noises were obviously decreased by up to 77.1% and 43.4% (*P* < 0.001). The periprosthetic soft tissues and bone evaluated were at best recognized with medium confidence on non-SEMAR images (median scores 2 ~ 4). When SEMAR was used, the image quality was significantly improved to high confidence (median scores 5, *P* < 0.001). The diagnostic confidence of prosthetic complications and tumor recurrence were also significantly increased by SEMAR (4 ~ 5 vs. 1 ~ 1.5, *P* < 0.001). Consequently, in this investigation, SEMAR could objectively and subjectively significantly mitigated the metal artifacts produced by not only titanium alloy but also cobalt-chrome-molybdenum alloy, and significantly increased the diagnostic confidence of prosthetic complications and tumor recurrence, which may play an important role in the follow-up of patients with knee tumor prostheses.

Virtual monochromatic images at high energy levels of the dual-energy CT had been confirmed to reduce the effects of beam hardening [22, 23]. However, bright and dark band artifacts caused by photon starvation from large metal and metal with high atomic numbers, such as total knee arthroplasty or the femoral stem of total hip arthroplasty, are too strong for dual-energy CT technique alone to sufficiently remove [16, 24]. Kidoh et al. reported that both the visual scores and signal-to-noise ratio were significantly higher for SEMAR than monochromatic images of dual-energy CT in patients with knee prostheses [10]. Additionally, the SEMAR algorithm can be applied to conventional single-energy CT with simple acquisition protocols and lower radiation exposure than dual-energy CT [9, 10], and doesn’t need careful pre-scan planning and can be applied retrospectively to routine volume data.

There are some limitations in this study. First, the number of patients with prosthetic complications and tumor recurrence was small. Second, considering the retrospective nature of this study, further prospective studies are needed to verify the effectiveness of SEMAR algorithm. Third, in most cases, the artifacts were not completely eliminated but substantially reduced by SEMAR. And projection-based MAR methods can generally introduce some kind of new artifacts. A previous study conducted by Andersson KM et al. [15] indicated that compared with SEMAR plus IR, SEMAR with filtered backprojection (FBP) created less new artifacts on CT imaging of hip prostheses. However, for some ROIs, the SEMAR plus IR images reconstructed with a soft kernel showed greater reduction of noise than FBP. With regard to the bone reconstruction kernel, FB in association with SEMAR, the image noise increased. Nevertheless, in this study, the artifacts from knee tumor prostheses were much stronger than other metal implants in previous studies, so the new artifacts produced by SEMAR were negligible.

## Conclusions

Compared with conventional CT images, the SEMAR algorithm significantly decreased the artifacts produced by not only photon starvation but also beam hardening. It improved both the objective and subjective image quality in patients with modular knee tumor prostheses, and increased diagnostic confidence of prosthetic complications and tumor recurrence near the metallic implants, especially at the articular level. So SEMAR could be recommended for post-operational follow-up of patients with knee tumor prosthesis. However, the artifacts were not completely eliminated by SEMAR and it might introduce slight new artifacts into the reconstructed images. In clinical practice, if necessary, the two data sets can be reconstructed and compared simultaneously to avoid such pitfalls.

## Data Availability

The dataset supporting the conclusions of this article is included within the article. Data and materials during the current study are available from the corresponding author upon reasonable request.

## Abbreviations

SEMAR: single energy metal artifact reduction
MDCT: multi-detector computed tomography
IR: iterative reconstruction
SD: standard deviations
CT: computed tomography
MAR: metal artifact reduction
HU: Hounsfield unit
AI: artifact index
ROI: region of interest
ICC: intra-class correlation coefficient
